# Reading profiles in secondary school: concurrent language and cognitive abilities, and retrospective and prospective reading skills

**DOI:** 10.3389/fpsyg.2023.1287134

**Published:** 2024-01-19

**Authors:** Christian Waldmann, Maria Levlin

**Affiliations:** ^1^Department of Swedish, Linnaeus University, Växjö, Sweden; ^2^Department of Language Studies, Umeå University, Umeå, Sweden

**Keywords:** cognitive abilities, language abilities, poor decoding, poor comprehension, Simple View of Reading, primary school, secondary school, upper-secondary school

## Abstract

**Introduction:**

We examined the concurrent language and cognitive abilities in a group of Swedish students with different reading profiles in secondary school, and the retrospective (primary school) and prospective (upper-secondary school) reading skills of each reading profile.

**Methods:**

Seventy-nine students participated in data collections in primary (grade 2: age 8), secondary (grade 8: age 14) and upper-secondary school (year 2: age 17). Independent variables included measures of word recognition, and vocabulary and text comprehension in secondary school. Dependent variables included measures of phonemic awareness, verbal fluency, listening comprehension, spelling, verbal working memory and nonverbal reasoning skills in secondary school, and word recognition and reading comprehension in primary and upper-secondary school.

**Results:**

When exploring the concurrent language and cognitive abilities of the reading profiles in secondary school, spelling emerged as a weakness and listening comprehension as a strength for students with poor decoding. Students with poor comprehension experienced weaknesses in spelling, and non-verbal reasoning. Students with both poor decoding and comprehension displayed a multi-deficit profile in language and cognition. As regards the retrospective and prospective reading skills, the relative ranking of the reading profiles was rather consistent in both primary and upper-secondary school.

**Discussion:**

The findings suggest that limitations in phonological awareness may not be a prominent feature of secondary school students with poor decoding in more transparent orthographies. From an educational perspective, spoken sources may support learning among students with poor decoding, whereas students with poor comprehension or combined difficulties in decoding and comprehension need support when learning from both spoken and written sources.

## Introduction

Reading comprehension is fundamental for school achievement, employment and participation in society (Ricketts et al., 2014; Holopainen et al., 2017; Levlin et al., 2022). The importance of developing reading comprehension in school is emphasized by research devoted to examining the language and cognitive abilities underlying reading comprehension and the development of reading comprehension during schooling. Much of this research has been guided by the Simple View of Reading (SVR) which identifies word recognition and language comprehension as the main constituents in developing reading comprehension (Hoover and Gough, 1990; Tunmer and Greaney, 2010; Tunmer and Hoover, 2019). Tunmer and Hoover (2019) define word recognition as word reading accuracy and fluency, and language comprehension as “the ability to extract and construct literal and inferred meaning from linguistic discourse represented in speech” (p. 78). Individual variations in word recognition and language comprehension may explain the developmental trajectory of reading comprehension and the various reading profiles that can be identified among students with reading difficulties. The SVR predicts that reading difficulties may be the result of difficulties in word recognition and/or language comprehension, leading to three different reading profiles: poor decoding (poor word recognition and age-typical language comprehension), poor comprehension (age-typical word recognition and poor language comprehension), and poor decoding and comprehension (poor word recognition and poor language comprehension) (e.g., Gough and Tunmer, 1986; Nation, 2019). Previous studies on the language and cognitive abilities underlying poor decoding and poor comprehension and the longitudinal development of poor decoding and poor comprehension have mainly focused on the early school years, shorter time periods and the most opaque (e.g., English) or most transparent (e.g., Finnish) orthographies. The current study extends previous research by exploring the underlying language and cognitive abilities among the reading profiles predicted by the SVR at the end of compulsory schooling, and the longitudinal development of reading skills among the reading profiles from primary to upper-secondary school in a semi-transparent orthography (Swedish).

## Background

### Language and cognitive abilities underlying the reading profiles poor decoding and poor comprehension

Word recognition and language comprehension are based on different underlying language and cognitive abilities and contribute uniquely and independently to reading comprehension (e.g., Hoover and Gough, 1990; Lervåg et al., 2018; Sleeman et al., 2022). Students with poor decoding usually experience difficulties in phonological processing, that is with storing (phonological short-term memory), retrieving (rapid automatic naming, RAN, and verbal fluency), and manipulating (phonemic awareness) phonological representations of words (e.g., Norrelgen et al., 2002; Catts et al., 2006; Melby-Lervåg et al., 2012; Elwér et al., 2013; Ramus et al., 2013). Phonemic awareness mainly affects students' abilities to learn grapheme-phoneme correspondence rules, that is word recognition accuracy (Melby-Lervåg et al., 2012; see also Brysbaert, 2022). Serial order short-term memory is associated with blending phonemes into words when decoding and may influence the learning of detailed orthographic representations necessary for efficient sight-word recognition and spelling (e.g., Melby-Lervåg et al., 2012; Nation and Castles, 2017; Ordonez Magro et al., 2020; Kemp and Treiman, 2022). Rapid retrieval of phonological information on the other hand is mainly associated with word recognition speed (Landerl et al., 2019). Difficulties in phonological processing may therefore explain the long-lasting challenges in word recognition and spelling regarding accuracy and fluency in students with poor decoding (Shaywitz et al., 1999; Puranik et al., 2007; Kairaluoma et al., 2013; Sumner et al., 2013; Diamanti et al., 2018).

While phonological processing usually emerges as a strength in students with poor comprehension (see Sleeman et al., 2022, for a different outcome in young students with poor comprehension), their poor language comprehension has been shown to affect both listening and reading comprehension across ages (e.g., Catts et al., 2006, 2016; Nation et al., 2010; Elwér et al., 2013; Sleeman et al., 2022). Poor language comprehension often includes limitations in vocabulary, comprehension of morphology and syntax, as well as discourse-level processes (Nation et al., 2004, 2010; Tunmer and Hoover, 2019). Furthermore, inferencing skills, verbal working memory and attention are co-occurring challenges (e.g., Justice et al., 2013; Cain and Bignell, 2014; Lervåg et al., 2018). Although age-typical non-verbal reasoning skills have typically been noticed among students with poor comprehension, there is still usually a significant discrepancy in non-verbal reasoning compared to age-matched controls (Nation et al., 2002, 2010; Catts et al., 2006).

The Simple View of Reading has proved to be stable and valid across school ages and different orthographies (Catts, 2018), and different studies have found that word recognition and language comprehension may explain between 40 and 99% of the variance in reading comprehension (e.g., Hoover and Gough, 1990; Malatesha Joshi and Aaron, 2000; Lervåg et al., 2018; Lonigan et al., 2018; Hjetland et al., 2019; Sleeman et al., 2022). However, there are cross-linguistic differences in developmental trajectories regarding the relative contribution of each component. In opaque orthographies, such as English, word-recognition (in particular accuracy) is more influential than language comprehension in the early school years, and language comprehension becomes more important in later stages of reading comprehension (Florit and Cain, 2011; Lonigan et al., 2018). In transparent orthographies, such as Finnish and Italian, oral language comprehension is the main predictor of reading comprehension already in primary school (e.g., Florit and Cain, 2011; Tobia and Bonifacci, 2015; Torppa et al., 2016; Cadime et al., 2017). The same pattern has been found in semi-transparent orthographies, for example in a study by Tapia Montesinos et al. (2022) with Spanish primary school students. This observed difference between opaque and (semi-)transparent orthographies has been attributed to differences in early decoding development; children learning to read (semi-)transparent orthographies reach a high degree of word recognition accuracy earlier than children in opaque orthographies (Seymour et al., 2003).

Furthermore, word recognition accuracy and fluency contribute differently to reading comprehension depending on orthographic transparency (e.g., Adlof et al., 2006; Language and Reading Research Consortium, 2015; Torppa et al., 2016). In opaque orthographies, accuracy emerges as a more significant contributor to reading comprehension than fluency in the earliest school ages; fluency becomes more influential from grade 3 and onwards (Georgiou et al., 2009; Language and Reading Research Consortium, 2015). By contrast, fluency is more influential already in grade 1 in transparent orthographies (Florit and Cain, 2011).

### Longitudinal development of reading skills

Although some children may develop poor word recognition and/or poor language comprehension after performing age-typically in the early school years (e.g., Catts et al., 2012), different types of reading difficulties typically emerge early and show stability and persistence over time in both opaque and (more) transparent orthographies (e.g., Juel, 1988; Jacobson, 1998; Catts et al., 2003, 2006; Cain and Oakhill, 2006; Svensson and Jacobson, 2006; Nation et al., 2010; Fouganthine, 2012; Elwér et al., 2013; Justice et al., 2013). Retrospectively, students with poor decoding and students with poor comprehension at different ages tend to have a history of poor word recognition and language comprehension, respectively (e.g., Catts et al., 2006; Elwér et al., 2013; Justice et al., 2013). Nation et al. (2010) found that 8-year-old British students with poor comprehension struggled with language comprehension already at the age of 5 years and reading comprehension at the age of 6 years. In addition, their progress in reading comprehension between 6 and 8 years was minimal. Delays in language skills among students with poor comprehension can be observed as early as at the age of 3 years (Justice et al., 2013). In a study of 926 American children in fourth grade, Elwér et al. (2013) found that students with poor decoding struggled with word recognition and spelling, and students with poor comprehension with vocabulary, morphology, grammar and listening comprehension, already in kindergarten. A similar pattern is found for secondary school students. Catts et al. (2006) found that students with poor decoding in grade 8 had poor word recognition skills but age-typical language comprehension in grade 2. Eighth-grade students with poor comprehension struggled with language comprehension but not with word recognition in kindergarten, supporting that phonological processing skills are usually intact in students with poor language comprehension (e.g., Stothard and Hulme, 1995; Cain et al., 2000; Nation et al., 2010). Typical readers, on the other hand, tend to have stable, age-typical word recognition and language comprehension already in the earliest grades (Catts et al., 2006; Nation et al., 2010).

Prospectively, poor word recognition and language comprehension in the early school years tend to persist during schooling (e.g., Juel, 1988; Jacobson, 1998; Cain and Oakhill, 2006) and into adulthood (Fouganthine, 2012). Jacobson (1998) studied a semi-transparent orthography, Swedish, and found that only a minority of students with poor decoding in second grade had achieved age-typical word recognition skills at the end of compulsory schooling. Fouganthine (2012) followed up the students with poor decoding in Jacobson (1998) at the age of 29–30 years and found that their word recognition skills had not developed much after grade 9. Some even had poorer word recognition skills in adulthood than in grade 9. Similarly, Cain and Oakhill (2006) found that British students with poor comprehension in grade 3 (7–8 years old) had persisting comprehension difficulties in grade 6 (10–11 years old), whereas students with typical comprehension maintained their good comprehension. Despite poor comprehension, the children had stable age-typical word recognition skills from grade 3–6.

## The current study

This study extends existing research in two ways. First, previous longitudinal studies have mainly focused on shorter time periods early in schooling, and only a few studies cover end of compulsory schooling and beyond. The current study investigates a longer time period, from grade 2 in primary school to year 2 in upper-secondary school. Further, by taking our starting-point in grade 8, we investigate the concurrent language and cognitive abilities among students with different reading profiles at an older age than in most previous studies, as well as the students' reading skills retrospectively in primary school and prospectively in upper-secondary school.

Second, whereas research focusing on opaque orthographies has a long tradition, studies on more transparent orthographies are fewer. Although the literature base in transparent orthographies has grown substantially in recent years, much research has focused on the most opaque or most transparent orthographies and paid less attention to languages with, for example, semi-transparent orthographies. This study explores the underlying language and cognitive abilities associated with poor decoding and comprehension, respectively, and the longitudinal development of reading skills among the different reading profiles in Swedish, which has a semi-transparent orthography.

The aim is (1) to investigate the language and cognitive abilities in students with different reading profiles at the end of compulsory schooling, and (2) to explore the students' prior and future reading skills. The following research questions are addressed:

(1) What are the similarities and differences between the reading profiles as regards concurrent measures of phonemic awareness, verbal fluency, listening comprehension, spelling, verbal working memory and non-verbal reasoning?
(2) How do the reading profiles perform on measures of word recognition and reading comprehension in grade 2 in primary school and year 2 in upper-secondary school?

## Methods

### Participants

One hundred thirty-two students were invited to participate at two different occasions during schooling, in eighth grade in secondary school and in their second year in upper-secondary school. The participants gave the researchers access to literacy measures collected in grade 2 in primary school (age 8), grade 8 in secondary school (age 14) and year 2 in upper-secondary school (age 17). In total, 97 students (50 girls) agreed to contribute data from primary and secondary school, and of them 79 students (35 girls) agreed to also contribute data from upper-secondary school. In other words, 79 students participated in data collections at all three time points.

The participating schools are placed in a municipality in a rural area in Sweden. The municipality has a slightly lower educational level than the national average (the rate of citizens with a post-upper-secondary degree is ~10% lower than the average in Sweden), while the unemployment rate is slightly below the national average (Ekonomifakta.se, 2022). There is only one secondary and upper-secondary school in the municipality. In upper-secondary school, the participating students were attending vocational (*n* = 43) as well as higher education preparatory programs (*n* = 36). All students had Swedish as their first language.

### Measures

The following measures were used to establish the reading profiles in grade 8 in secondary school (independent variables):

#### Word recognition

A composite measure of phonological decoding and orthographic word recognition (Olofsson, 1998) was used. Both tasks involve word recognition accuracy and fluency. In the phonological decoding task, the students read triplets of pseudo-words silently and marked the pseudo-word sounding like a real word, for example *vasp*—*jus*—*sorf* (*jus* is a homophone to *ljus* “light”). The score was the number of correctly marked homophones identified within 2 min. In the orthographic word recognition task, the students read pairs of words silently. One word in each pair was correctly spelled, and one was a pseudo-homophone, for example *taksi*-*taxi*. Students were asked to mark the correctly spelled word in as many word pairs as possible within 2 min. The score was the number of correctly marked words. Raw scores were converted to standardized scores (*z*-scores) using the normative means and SDs in the manual. The composite measure was the mean of the *z*-scores for phonological decoding and orthographic word recognition. Cronbach's alpha of the composite measure was 0.78.

#### Comprehension

In previous studies on the SVR, measures of comprehension have been based on either listening (Catts et al., 2003, 2005) or reading comprehension (Nation et al., 2004, 2010; Cain and Oakhill, 2006) tasks. Since group assessments were used in this study, we decided to follow Nation with colleagues and use a composite reading comprehension measure based on text comprehension (Johansson, 2004) and vocabulary comprehension (Järpsten, 2002). In the text comprehension task, students read silently nine short texts within 35 min. After each text followed six statements capturing literal as well as inferential content. Students were asked to identify the statements that were consistent with the text content as well as choosing a title for the text from five alternatives. The score was the number of correctly marked statements and titles minus the number of incorrectly marked ones (maximum 36 points). The internal validity was 0.83 (Johansson, 2004). In the vocabulary comprehension task, students read silently a target word and then identified a synonym among four alternatives. Thirty-four target words were included, and the time limit was 20 min. The score was the number of correctly marked synonyms. The internal validity was 0.78 (Järpsten, 2002). The conversion to *z*-scores was based on the normative means and SDs in the manuals. The composite measure was the mean of the *z*-scores for text and vocabulary comprehension. Cronbach's alpha of the composite measure was 0.70.

The following measures were used to examine the language and cognitive profiles of the reading profiles in grade 8 in secondary school (dependent variables):

#### Phonemic awareness—spoonerisms

The students listened to a word pair with swapped initials, for example *mund råne* for *rund måne* “round moon”. They were asked to swap the initials back, and to identify a picture out of three alternatives that was describing the real word pair. The task had no time limit. This was an adjustment to the age-group and a deviation from the normal procedure. Twenty-four spoonerisms were included, and the score was the number of correctly identified pictures (maximum 24 points). *Z*-scores were calculated based on the normative mean and SD in the manual (Lundberg and Wolff, 2003). Reported test-retest reliability was 0.68.

#### Verbal fluency

A composite measure of semantic and phonemic fluency was used (Carlsson, 2009, see also Henry et al., 2015). Following Unsworth et al. (2011), students were asked to provide their answers in writing. In the semantic fluency task, students wrote as many words for animals as possible within 1 min. In the phonemic fluency task, the students wrote as many words with the initial letters *f* , *a*, and *s* as possible. The time limit was 1 min per letter. The score for each task was the number of correct words. Raw scores were converted to *z*-scores using the normative means and SDs in the manuals. The composite measure was the mean of the *z*-scores for semantic and phonemic fluency. Cronbach's alpha of the composite measure was 0.71.

#### Listening comprehension

The listening comprehension task was a modification of the re-telling task in Taube et al. (1984). The students listened to a story about a trip with an air-balloon and wrote down the story with their own words. The score was the number of correctly recollected information units in the students' written texts (maximum 26 points). As this task lacks norm-references, raw scores were converted to *z*-scores based on the mean and SD in a sample of 102 students in grade 8, which also included the students participating in the current study. No reliability measures are available.

#### Spelling

The students listened to sentences containing a target word. The target word was orally repeated by the test leader and written down by the students. Most target words had a rather complex phonological (several consonant clusters in the same word), orthographic (irregularities in spelling) and morphological (compound words) structure. The score was the number of correctly spelled words (maximum 50 points). *Z*-scores were calculated using the normative mean and SD in the manual (Johansson, 2004). Reported Cronbach's alpha was 0.89.

#### Verbal working memory

A consonant letter was orally presented to the students and immediately followed by two sentences. The first sentence was a statement, for example *Horses gallop in the paddock*, and the second sentence was a related question to the statement, for example *Can horses talk?* Each question required a response from the students with a written *yes*- or *no*-sign. The procedure continued with a new consonant and two more sentences in the same format. Finally, students were asked to write down the presented consonants in the correct order. The task included two trials with two consonants, two trials with three consonants and two trials with four consonants. Two points were given for a correct consonant with a correct position in the sequence, and one point was given for a correct consonant but in the wrong position (maximum 36 points). Raw scores were converted to *z*-scores using the normative mean and SD in the manual (Wolff, 2010). Reported Cronbach's alpha was 0.79.

#### Nonverbal reasoning skills

Nonverbal reasoning skills were assessed using an adapted and computerized version of Raven's (2000) progressive matrices. Students were asked to identify the missing element out of six alternatives to complete a pattern. The score was the number of correctly identified missing elements (maximum 12). As this task lacks norm-references, raw scores were converted to *z*-scores based on the mean and SD in the same sample as for the listening comprehension task (see above). No reliability measures are available.

The following measures were used to examine the students' word recognition and reading comprehension retrospectively in grade 2 in primary school and prospectively in year 2 in upper-secondary school (dependent variables).

#### Word recognition

A composite measure of phonological decoding and orthographic word recognition (Olofsson, 1998) was used in both primary and upper-secondary school. The tasks, items, procedures and scoring were the same as in grade 8 (see above). As the tasks lack norm-references for primary school, the raw scores were converted to *z*-scores using the means and SDs in a sample of 187 students in grade 2, which also included the students in the current study. *Z*-scores for upper-secondary school were calculated based on the normative means and SDs in the manual. The composite measures were the means of the *z*-scores for phonological decoding and orthographic word recognition in primary and upper-secondary school respectively. Cronbach's alpha of the composite measure was 0.78 for primary and 0.79 for upper-secondary school.

#### Reading comprehension

In primary school, the students silently read short paragraphs of text followed by a multiple-choice task with four alternatives capturing mostly literal content of the text. The score was the number of correct answers within 30 min (maximum 18 points). In upper-secondary school, the students silently read three factual texts. Each text was followed by a multiple-choice task capturing both literal and inferential content of the text. The time-limit for the task was 35 min. The score was the number of correct answers (maximum 21 points). Raw scores were converted to *z*-scores using the normative means and SDs in the manuals for primary and upper-secondary school. Reported Cronbach's alpha was 0.62 and 0.74 for primary (Järpsten and Taube, 1999) and upper-secondary school (Järpsten and Taube, 2017), respectively.

### Procedure

The study was conducted in accordance with the Swedish Ethical Review Act relating to research involving humans SFS (2003:460, 2003) and the ethics guidelines of the Swedish Research Council (2017). The project was assessed by the Regional Ethical Review Board in Umeå, Sweden, as not falling under the Swedish Ethical Review Act. Informed consent was obtained from parents, students and schools prior to the assessments in grade 8 in secondary school and year 2 in upper-secondary school. The assessment tasks were carried out in groups in the classroom by the teachers in primary school (part of the regular routines in the municipality) and by the teachers in collaboration with the research team in secondary and upper-secondary school. The administration of the tasks followed the standard procedures in the manuals if nothing else is reported in the description of the measures (see above). The students were allowed to terminate the assessments at any time. Some students were absent from some sessions.

The model Simple View of Reading was used to identify four reading profiles (typical reading, poor decoding, poor comprehension, and poor decoding and comprehension) based on the students' word recognition and comprehension scores in grade 8 in secondary school. The cut-off for poor word recognition and poor comprehension was set at *z* ≤ −0.70, and for typical word recognition and comprehension at *z* ≥ −0.69. We used the same cut-off to define typical and poor performance on all dependent variables. In other words, we define performance within typical range on all measures as scoring *z* ≥ −0.69. We decided on a more lenient cut-off score than what is typically used when defining, for example, dyslexia (*z* ≤ −1.0), since there is some evidence that performance below more lenient cut-offs may have long-lasting effects on educational attainment (see Levlin et al., 2022) and persist over time (Catts et al., 2006).

**Figure 1:**
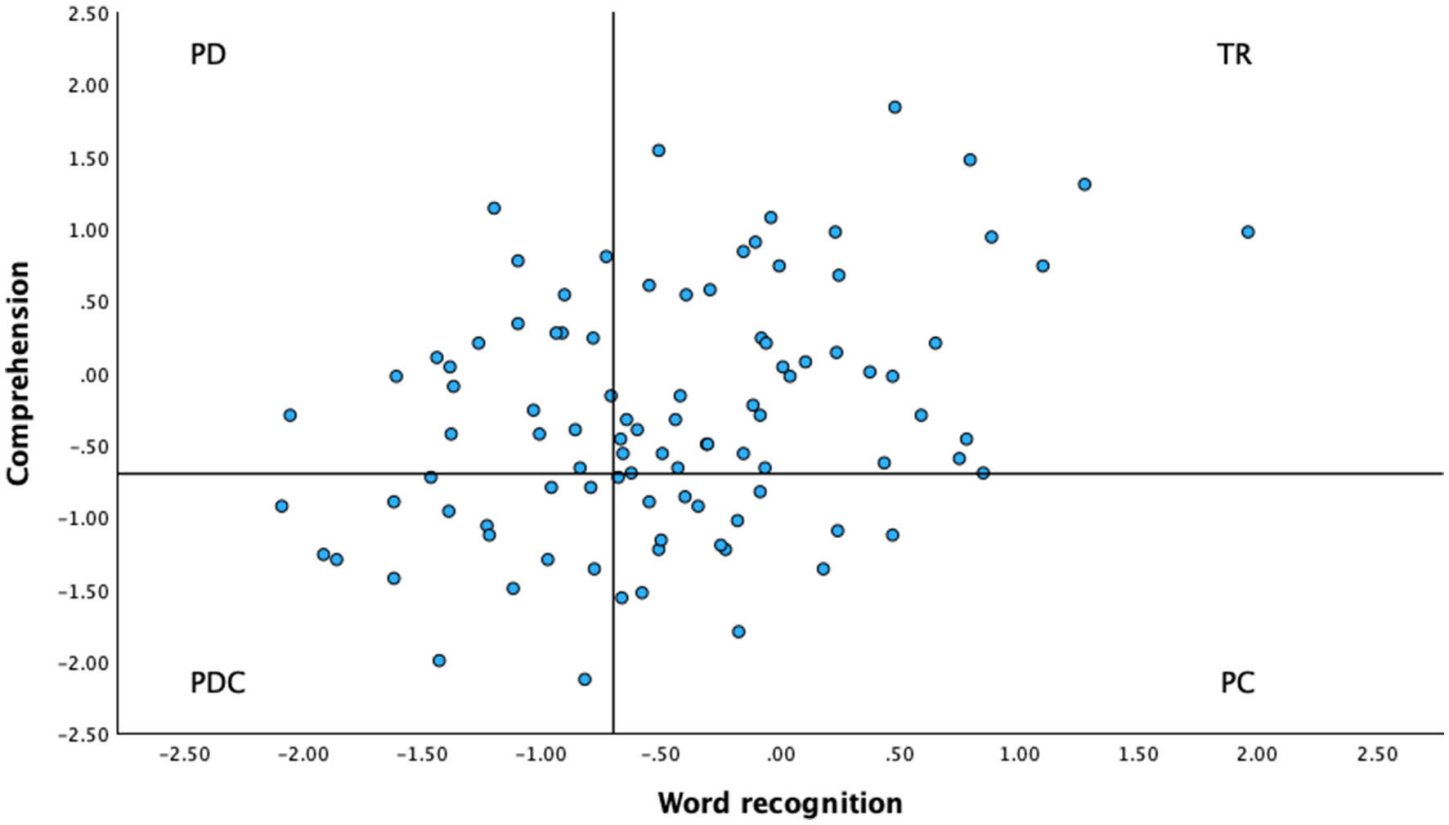
The distribution of students (*N* = 97) on four reading profiles (TR, typical reading; PD, poor decoding; PC, poor comprehension; PDC, poor decoding and comprehension) based on their performance (*z*-scores) on measures of word recognition and comprehension in secondary school (grade 8).

Twenty students scored below cut-off (*z* ≤ −0.70) on word recognition only and were classified as students with poor decoding (PD). Eighteen students scored below cut-off on comprehension only and were classified as students with poor comprehension (PC). Sixteen students scored below cut-off on both word recognition and comprehension and were classified as students with poor decoding and comprehension (PDC). The remaining 43 students had typical reading skills (TR); they scored above cut-off (*z* ≥ −0.69) on both word recognition and comprehension. Figure 1 shows the distribution of the students across the four reading profiles. Means and SDs of students with TR, PD, PC, and PDC on word recognition and comprehension are presented in Table 1.

**Table 1:** Performance (*z*-scores) of students with typical reading (TR), poor decoding (PD), poor comprehension (PC) and poor decoding and comprehension (PDC) on measures of word recognition and comprehension in secondary school (grade 8).

	**TR (*n* = 43)**	**PD (*n* = 20)**	**PC (*n* = 18)**	**PDC (*n* = 16)**	**TOTAL (*n* = 97)**
	**Mean (SD)**	**Mean (SD)**	**Mean (SD)**	**Mean (SD)**	**Mean (SD)**
Word recognition	0.09 (0.58)	−1.13 (0.34)	−0.22 (0.42)	−1.33 (0.41)	−0.46 (0.77)
Comprehension	0.20 (0.69)	0.10 (0.47)	−1.11 (0.31)	−1.22 (0.41)	−0.30 (0.84)

As can be seen in Table 1, the mean values in word recognition and comprehension for all participants are slightly below the normative means for the age-group in the manuals. There is also a higher number of students identified with poor word recognition and/or poor comprehension than expected in relation to the cut-off (*z* ≤ −0.70).

### Data analyses

Descriptive and non-parametric statistics were calculated using the Statistical Package for the Social Sciences (IBM SPSS, version 24). An initial exploration of the descriptive data and the boxplots for all measures revealed outliers in word recognition in primary (*n* = 1), secondary (*n* = 1) and upper-secondary school (*n* = 2), in phonemic awareness (*n* =3), verbal fluency (*n* = 2), listening comprehension (*n* = 3) and working memory (*n* = 1) in secondary school, and in reading comprehension (*n* = 2) in upper-secondary school. All outliers were corrected to the nearest value not identified as an outlier, following the procedure described in Field (2013, p. 198). Further explorations of the data, including analyses of variance, revealed violations of normality (Shapiro-Wilk) and homogeneity of variances (Levene) in several measures. Shapiro-Wilk statistics showed significant results for word recognition in primary school (*p* < 0.001), for phonemic awareness (*p* < 0.001), listening comprehension (*p* = 0.035), working memory (*p* < 0.001) and non-verbal reasoning skills (*p* = 0.002) in secondary school, and for reading comprehension in upper-secondary school (*p* < 0.001). An inspection of the histograms revealed floor effects for word recognition in primary school. Ceiling effects were found for phonemic awareness and working memory in secondary school, and for reading comprehension in upper-secondary school. Levene's test for homogeneity of variances showed significant results for word recognition in primary school (*p* < 0.001), for phonemic awareness (*p* = 0.031) and working memory (*p* < 0.001) in secondary school, and for reading comprehension in upper-secondary school (*p* = 0.039).

Therefore, we used the Kruskal-Wallis H Test with *post-hoc* comparisons to analyse differences between reading profiles in retrospective (primary school), concurrent (secondary school) and prospective (upper-secondary school) measures of reading, language and cognitive abilities. The Mann-Whitney U-Test was used for multiple *post-hoc* comparisons between reading profiles. The Wilcoxon Signed Rank Test was used to assess the development of reading from grade 2 in primary school to year 2 in upper-secondary school for each reading profile. The magnitude of significant differences was calculated using eta-squared (small effect = ≤ 0.05, medium effect = 0.06–0.13, large effect = ≥0.14 (Cohen, 1988). The significance level was set at 95% (*p* < 0.05). When conducting the Mann-Whitney U-Test, we selected automatic Bonferroni adjustment to compensate for multiple comparisons.

## Results

### RQ1: concurrent language and cognitive abilities in secondary school

Table 2 shows the scores for the four reading profiles on all concurrent measures of language and cognitive abilities in grade 8 in secondary school. There were significant group differences on phonemic awareness [*H* (3, *n* = 89) = 16.15, *p* = 0.001, η^2^ = 0.184], verbal fluency [*H* (3, *n* = 85) = 10.33, *p* = 0.016, η^2^ = 0.123], listening comprehension [*H* (3, *n* = 77) = 10.50, *p* = 0.015, η^2^ = 0.138], spelling [*H* (3, *n* = 90) = 32.51, *p* < 0.001, η^2^ = 0.365] and nonverbal reasoning [*H* (3, *n* = 85) = 22.45, *p* < 0.001, η^2^ = 0.267] with medium to large effect sizes for all measures. Verbal working memory [*H* (3, *n* = 91) = 5.26, *p* = 0.153, η^2^ = 0.058] did not reach significance and displayed a small to medium effect size.

**Table 2:** Performance on measures of phonemic awareness, verbal fluency, listening comprehension, spelling, working memory, and non-verbal reasoning by reading profile.

	**TR (*n* = 41)**	**PD (*n* = 20)**	**PC (*n* = 18)**	**PDC (*n* = 15)**	**Group differences (Kruskal-Wallis)**
	**Mean (SD)**	**Mean (SD)**	**Mean (SD)**	**Mean (SD)**	
Phonemic awareness	0.36 (0.79)	0.07 (0.75)	−0.39 (1.21)	−0.71 (1.21)	TR > PDC
Verbal fluency	0.33 (0.84)	−0.27 (0.85)	−0.16 (0.71)	−0.48 (0.89)	
Listening comprehension	−0.01 (0.86)	0.59 (0.74)	−0.38 (1.08)	−0.67 (1.27)	PD > PDC
Spelling	0.29 (0.76)	−0.63 (0.83)	−0.91 (0.78)	−1.13 (0.96)	TR > PD/PC/PDC
Verbal working memory	0.24 (0.74)	0.05 (0.86)	0.07 (0.91)	−0.82 (1.46)	
Non-verbal reasoning	0.29 (0.85)	0.46 (1.16)	−0.79 (0.76)	−0.63 (0.66)	TR/PD > PC/PDC

Pairwise comparisons showed that students with TR performed significantly better than students with PDC on phonemic awareness (*p* = 0.001). Students with PD performed significantly better on listening comprehension than students with PDC (*p* = 0.021). Students with TR performed significantly better on spelling than students with PD (*p* = 0.002), PC (*p* < 0.001) and PDC (*p* < 0.001). Regarding non-verbal reasoning, students with TR and students with PD performed significantly better than students with PC (*p* = 0.003 and *p* = 0.001, respectively) and students with PDC (*p* = 0.025 and *p* = 0.009, respectively). No other significant differences between the reading profiles were found.

### RQ2: retrospective (primary school) and prospective (upper-secondary school) word recognition and reading comprehension

Table 3 presents the scores on retrospective measures of word recognition and reading comprehension in grade 2 in primary school by eighth-grade reading profile. There were significant group differences on word recognition [*H* (3, *n* = 94) = 22.65, *p* < 0.001, η^2^ = 0.244] and reading comprehension [*H* (3, *n* = 94) = 22.76, *p* < 0.001, η^2^ = 0.245] with large effect sizes. Pairwise comparisons showed that students with TR performed significantly better on word recognition than students with PD (*p* = 0.001) and PDC (*p* = 0.001). As regards reading comprehension, students with TR scored significantly better than students with PC (*p* = 0.001) and PDC (*p* = 0.001). No other significant group differences were found.

**Table 3:** Performance on retrospective measures of word recognition and reading comprehension in primary school (grade 2) by eighth-grade reading profile.

	**TR (*n* = 42)**	**PD (*n* = 19)**	**PC (*n* = 18)**	**PDC (*n* = 15)**	**Group differences (Kruskal-Wallis)**
	**Mean (SD)**	**Mean (SD)**	**Mean (SD)**	**Mean (SD)**	
Word recognition	0.47 (0.97)	−0.53 (0.52)	−0.26 (0.76)	−0.63 (0.39)	TR > PD/PDC
Reading comprehension	0.56 (0.88)	0.04 (0.93)	−0.53 (1.04)	−0.55 (0.80)	TR > PC/PDC

Table 4 shows the scores on prospective measures of word recognition and reading comprehension in year 2 in upper-secondary school by eighth-grade reading profile. There were significant group differences on word recognition [*H* (3, *n* = 74) = 30.82, *p* < 0.001, η^2^ = 0.422] and reading comprehension [*H* (3, *n* = 75) = 13.86, *p* < 0.001, η^2^ = 0.187] with large effect sizes. Pairwise comparisons showed that students with TR performed significantly better on word recognition than students with PD (*p* = 0.001) and PDC (*p* = 0.001). As regards reading comprehension, students with PDC scored significantly worse than students with TR (*p* = 0.005) and PD (*p* = 0.011). No other significant group differences were found.

**Table 4:** Performance on prospective measures of word recognition and reading comprehension in upper-secondary school (year 2) by eighth-grade reading profile.

	**TR (*n* = 36)**	**PD (*n* = 16)**	**PC (*n* = 13)**	**PDC (*n* = 10)**	**Group differences (Kruskal-Wallis)**
	**Mean (SD)**	**Mean (SD)**	**Mean (SD)**	**Mean (SD)**	
Word recognition	0.60 (0.70)	−0.40 (0.66)	0.14 (0.57)	−0.93 (0.70)	TR > PD/PDC
Reading comprehension	0.54 (0.64)	0.58 (0.57)	0.17 (0.58)	−0.80 (1.21)	TR/PD > PDC

**Figure 2:**
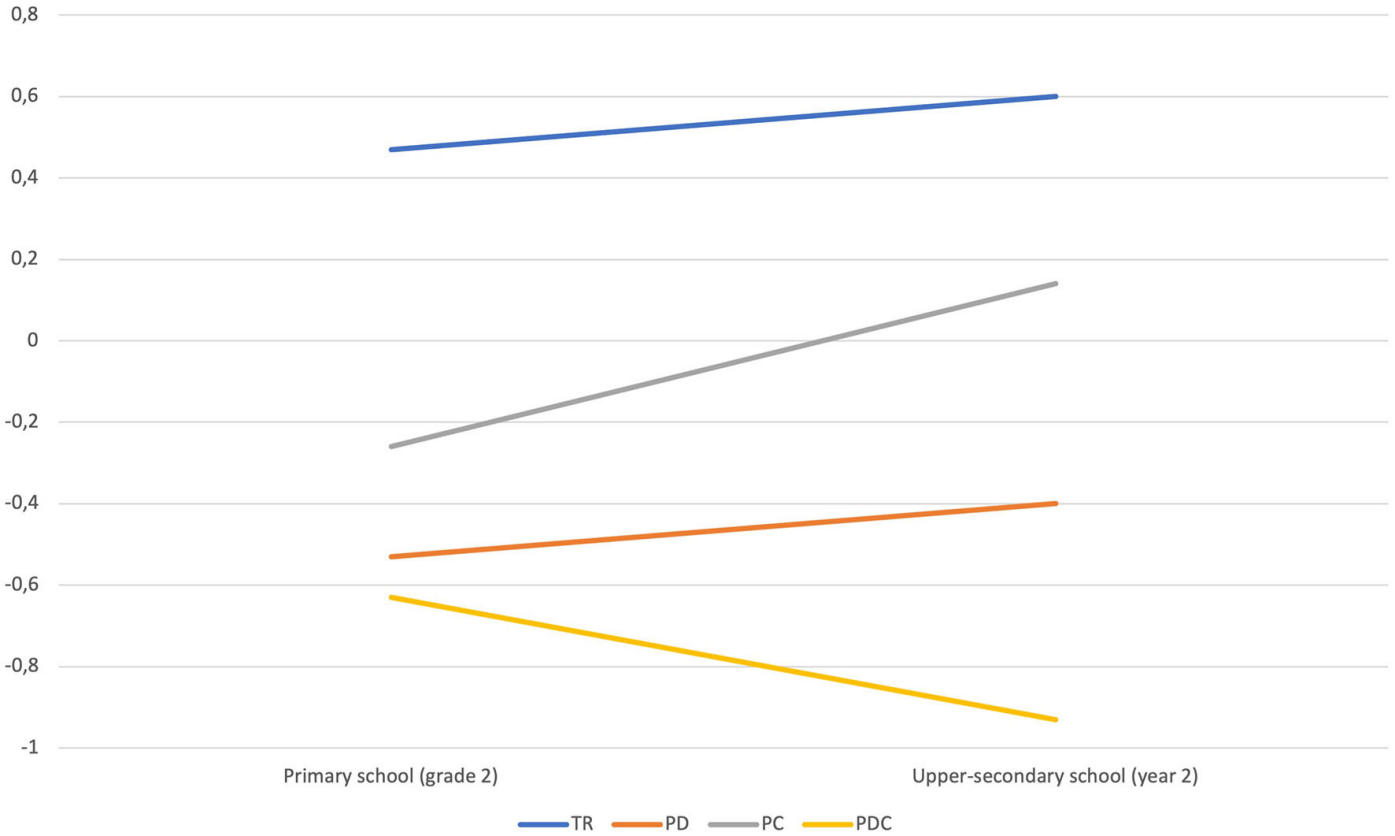
Word recognition scores (means) in primary school (grade 2) and upper-secondary school (year 2) by eighth-grade reading profile.

**Figure 3:**
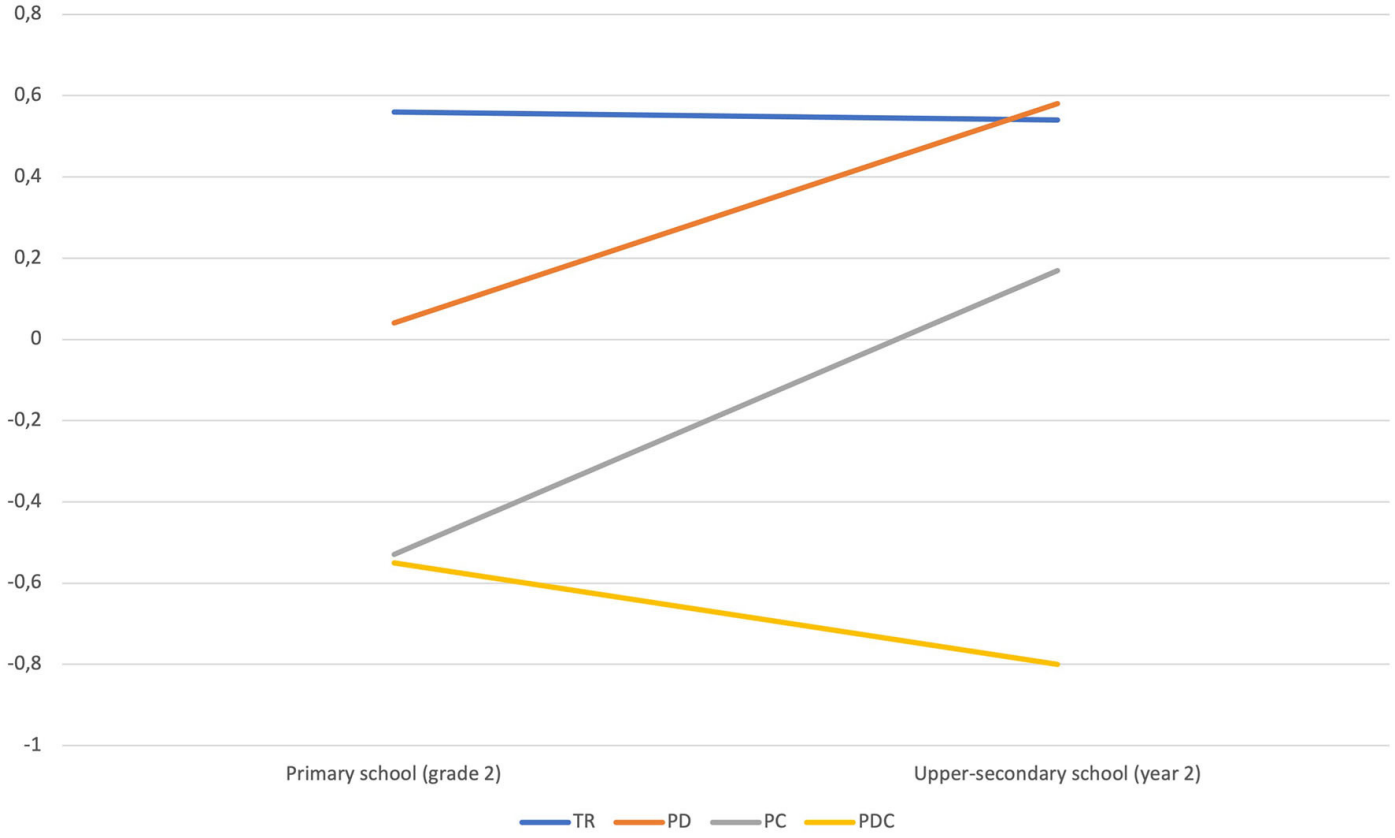
Reading comprehension scores (means) in primary school (grade 2) and upper-secondary school (year 2) by eighth-grade reading profile.

Figures 2 and 3 illustrate the change in scores on word recognition and reading comprehension from grade 2 in primary school to year 2 in upper-secondary school by eighth-grade reading profile. The figures show that all reading profiles in grade 8, except students with PDC, scored higher on word recognition and reading comprehension in upper-secondary school than they did in primary school. A Wilcoxon Signed Rank Test was conducted to assess if changes in scores from primary to upper-secondary school were significant for each reading profile. Word recognition scores did not increase significantly from primary to upper-secondary school for students with TR (*p* = 0.351), PD (*p* = 0.255), and PC (*p* = 0.116). Reading comprehension scores increased significantly for students with PD (*p* = 0.038) and PC (*p* = 0.013), but not for students with TR (*p* = 0.706). In contrast to the other reading profiles, students with PDC in grade 8 scored lower on both word recognition and reading comprehension in upper-secondary school than they did in primary school. The decreases in scores from primary to upper-secondary school were not significant (*p* = 0.889 and *p* = 0.859).

## Discussion

This study examined (1) the concurrent underlying language (phonemic awareness, verbal fluency, listening comprehension and spelling) and cognitive (verbal working memory and non-verbal reasoning) abilities in students with the reading profiles typical reading (TR), poor decoding (PD), poor comprehension (PC), and poor decoding and comprehension (PDC) in grade 8 in secondary school, and (2) the retrospective (grade 2 in primary school) and prospective (year 2 in upper-secondary school) reading skills of each reading profile. The study revealed similarities and differences in concurrent language and cognitive abilities between the students in the four reading profiles. As expected, students with TR scored at or above mean for same-aged peers on all measures in grade 8. Students with PD also scored within typical range on all measures. They showed the strongest performance on listening comprehension of all reading profiles, which is in line with the predictions of SVR that poor decoders with a good reading comprehension also have a good listening comprehension. Thus, in educational settings, students with poor decoding may benefit from learning through listening.

However, students with PD performed significantly lower than students with TR in spelling. In line with much previous studies (e.g., Puranik et al., 2007; Sumner et al., 2013; Diamanti et al., 2018), this may indicate concurrent limitations in phonological processing (e.g., Melby-Lervåg et al., 2012; Kairaluoma et al., 2013; Torppa et al., 2017) or in detailed word-specific orthographic knowledge (Shahar-Yames and Share, 2008; Conrad et al., 2019; Querido et al., 2021). This study did not confirm such phonological limitations; students with PD performed on par with students with TR on both phonemic awareness and verbal fluency. One suggestion that has been advanced by Bishop (1997) is that students with poor decoding may suffer from the remains of a phonological deficit, and that tests in older age-groups are not sensitive enough to identify such remains. In addition, phonemic awareness is not very influential on word recognition in transparent orthographies, especially not after the initial stage of learning to read (Furnes and Samuelsson, 2011; Landerl et al., 2019). This may be due to the close grapheme-phoneme correspondence in transparent orthographies. However, RAN and verbal fluency continue to predict variance in reading speed in transparent orthographies in both younger (Landerl et al., 2019) and older age-groups (Kairaluoma et al., 2013). The measure of verbal fluency in this study taxed processing speed of semantic and phonological information. A measure of RAN based on automatized knowledge of digits may be more sensitive in capturing variation in processing speed than the verbal fluency measure used in this study. Future studies need to examine different aspects of phonological processing skills over time from kindergarten to upper-secondary school in order to explore their potential contribution in explaining individual variations in word recognition and spelling.

Alternatively, the poor outcome in word recognition and spelling in students with PD may indicate limitations in orthographic knowledge. As shown by for example Shahar-Yames and Share (2008) and Conrad et al. (2019), spelling requires an active retrieval of detailed orthographic knowledge, while word recognition requires the capacity to recognize spelling-sound patterns. Querido et al. (2021) explored the contribution of orthographic knowledge in grade 2 to word recognition and spelling in grade 3, as well as the contribution of orthographic knowledge in grade 4 to word recognition and spelling in grade 5 in Portuguese (semi-transparent), in two different cohorts, respectively. Orthographic knowledge contributed significantly to the variance in both word recognition and spelling in grade 3, and to spelling in grade 5. The importance of orthographic knowledge for spelling was also found in a study by Åsberg Johnels et al. (in press) with partly the same cohort of students as in this study. Orthographic knowledge in grade 2 contributed significantly to spelling scores in grade 8 when controlling for phonological recoding and the autoregressive effect of spelling in grade 2. This may indicate that orthographic knowledge plays a more important role than phonological knowledge in later school years, at least in semi-transparent orthographies.

Previous research on students with poor comprehension (PC) has identified phonemic awareness and spelling as relative strengths, and listening comprehension, verbal working memory and non-verbal reasoning as limitations (Nation et al., 2004, 2010; Catts et al., 2006; Adlof et al., 2010; Elwér et al., 2013; Sleeman et al., 2022), a pattern only partly confirmed in this study. Students with PC performed on par with students with TR on verbal fluency, verbal working memory, phonemic awareness and listening comprehension, and significantly lower than students with TR only on spelling and non-verbal reasoning. The weak outcome in non-verbal reasoning confirms that general reasoning skills can be a challenge for students with PC (Nation et al., 2002, 2010; Catts et al., 2006). An unexpected finding was the performance almost on par with students with TR in listening comprehension. This may be due to the construction of the listening comprehension task, taxing students' summarizing skills of a story line rather than their inferencing skills. Another type of listening comprehension task might have led to another outcome. This indicates that this group of students may cope well with listening and retelling stories or oral briefings containing every-day language in an educational context. However, when the assignment requires comprehension of more complex language and inferencing skills students with PC may need support when learning from both spoken and written sources, as is reflected in their poor outcome in the composite measure of vocabulary and reading comprehension in grade 8. As Tunmer and Hoover (2019) point out, students belonging to this reading profile need specialized instructions targeting underlying processes supporting comprehension, such as making inferences, activating relevant background knowledge, and vocabulary and grammar.

Another unexpected finding was that the students with PC experienced limitations in spelling, despite intact word recognition skills. This finding supports the idea of a dissociation between spelling and word recognition in more transparent orthographies (Furnes and Samuelsson, 2011; Landerl et al., 2019). Poor spelling in combination with good word recognition is unusual in opaque orthographies, but not uncommon in more transparent orthographies. As long as RAN/verbal fluency is spared, as was the case in this study for students with PC, word recognition in more transparent orthographies may follow a typically developing trajectory, despite concurrent limitations in spelling (see also Pennington and Bishop, 2009; Torppa et al., 2017).

Students with poor decoding and comprehension (PDC) performed relatively poor on all measures compared to the other reading profiles. They scored significantly lower than student with TR on phonemic awareness, spelling and non-verbal reasoning, and significantly lower than students with PD on listening comprehension and non-verbal reasoning. This is in line with a previous study by Sleeman et al. (2022) including 209 English-speaking students in primary school. The current study suggests that this pattern is still evident also in secondary school. In sum, these students experienced the collective limitations of students with PD and students with PC (cf., Nation, 2019), which potentially makes them highly vulnerable in educational settings. An educational implication is that students with PDC should be prioritized for special needs support.

The findings partly confirmed a similar relative ranking of the reading profiles identified in grade 8 in secondary school, retrospectively in primary school and prospectively in upper-secondary school (e.g., Juel, 1988; Jacobson, 1998; Catts et al., 2003, 2006; Cain and Oakhill, 2006; Svensson and Jacobson, 2006; Nation et al., 2010; Fouganthine, 2012; Elwér et al., 2013; Justice et al., 2013; Catts, 2018). Most mean values for word recognition and reading comprehension were within typical range in both primary and upper-secondary school, except for students with PDC in upper-secondary school. This indicates that students with poor reading skills in grade 8 have not necessarily experienced poor reading skills in primary school, and do not necessarily continue to experience poor reading skills in upper-secondary school. However, when considering the reading profiles' relative ranking, students with TR, PD, and PDC showed a similar relative ranking over time, while the developmental trajectory for students with PC was more varied.

The students with TR in grade 8 in secondary school had typical reading skills already in grade 2 in primary school and continued to have typical reading skills also in year 2 in upper-secondary school. Students with PD in secondary school performed significantly below students with TR on word recognition and at the same level as students with TR on reading comprehension in both primary and upper-secondary school. Their age-typical comprehension skills in secondary school were robust over time and not impeded by their difficulties in word recognition. To our knowledge, no previous studies have examined the development of reading comprehension for this group of students in upper-secondary school. The findings in this study showed that students with PD continued to cope even when the demands on inferential skills increase in reading comprehension tasks in upper-secondary school. It is possible, however, that there would be another outcome for this group of students in an opaque orthography, where the reading process places higher demands on word reading accuracy.

Students with PDC in secondary school performed significantly below students with TR on word recognition and reading comprehension in both primary and upper-secondary school. However, in primary school, their performance was within the typical range on both reading measures, albeit in the lower end, which indicates that the reading difficulties in secondary school may have been less evident in primary school, at least for some students. This aligns with Catts et al. (2006) who examined a group of students with PC only and found that language deficits in secondary school (grade 8) were less obvious in kindergarten. One explanation for our results may be that lower demands on word recognition and reading comprehension in the early school years may hide difficulties that are revealed with increasing demands later in schooling. Unfortunately, students with PDC showed a negative developmental trajectory after grade 8 with a slightly increasing gap compared to the other reading profiles in both word recognition and reading comprehension in upper-secondary school. The outcome in language and cognitive abilities in grade 8 in secondary school may explain this negative development. Students with PDC performed significantly below students with TR and PD on several measures, such as phonemic awareness, verbal working memory, listening comprehension and non-verbal reasoning. This indicates that students with PDC seem to have more of a multi-deficit profile in language and cognition compared to students with PD and PC.

The students with PC in secondary school performed within typical range on word recognition in primary and upper-secondary school and did not differ significantly from students with TR at any point in time. By contrast, their reading comprehension showed a more varied developmental trajectory. The comprehension difficulties in secondary school were evident also in primary school with reading comprehension scores significantly below students with TR. After grade 8, their reading comprehension developed positively, and in upper-secondary school they performed within typical range with no significant differences compared to students with TR and PD. This promising finding may be related to their outcomes in language and cognition in secondary school; they performed on par with the students with TR on all measures except spelling and non-verbal reasoning. Poor non-verbal reasoning may be a risk-factor impeding demanding tasks such as reading comprehension, but this does not seem to be the case in this group of students with PC. It is possible that their relative strengths in verbal working memory and verbal fluency in grade 8 explain their positive development and age-typical performance on reading comprehension in upper-secondary school. They are not hindered by a multi-deficit profile as is the case for students with PDC. To our knowledge, no previous studies have explored the reading development of students with PC from primary to upper-secondary school. In previous studies, it has, however, been found that students with PC reached the same levels in educational attainment as controls at the end of compulsory schooling (age 16), despite significantly lower performance at age 11 (Ricketts et al., 2014). Future studies need to examine the development in both comprehension and educational attainment for students with PC in adolescence and post-compulsory schooling.

## Limitations

The longitudinal approach covering primary to upper-secondary school in combination with the grouping of students into different reading profiles presents some limitations. Firstly, attrition poses a challenge to all longitudinal studies, and highlights the need to recruit enough participants to ensure a high number of participants throughout the whole period of investigation. The longer a study proceeds, the more participants tend to drop out, reducing the initial sample. In this study, we followed students over a 10-year time period from the age of 8–17 years, covering the main part of the students' compulsory and voluntary schooling. The rather small sample size and the observed violations of normality and homogeneity of variances in the data limit the choice of statistical analyses and the magnitude of the statistical analyses that we have performed. Thus, our findings need to be interpreted with caution.

Secondly, selecting measures for a longitudinal study covering almost 10 years was challenging. A significant limitation of the study design is that we did not have access to the same measures of language and cognitive abilities in primary and upper-secondary school as in secondary school. Such data would have contributed valuable knowledge about the longitudinal development of the students' language and cognitive abilities from primary to upper-secondary school. A related issue concerns the lack of norm-references for measures of word recognition in primary school and listening comprehension and non-verbal reasoning in secondary school. This significantly limited our interpretations of findings based on these measures. A norm-referenced language comprehension measure based on listening rather than reading would have been more appropriate for the identification of students with different reading profiles in grade 8. As regards students with poor comprehension and age-typical word recognition (PC), it is plausible to conclude that their poor outcome in comprehension was related to limitations in language comprehension and not to limitations in word recognition skills. By contrast, it is more difficult to disentangle to what extent the poor comprehension in students with poor decoding and comprehension (PDC) was related to limitations in language comprehension, or whether their performance on the comprehension task was also influenced by their poor word recognition skills. Also, the concurrent outcomes in listening comprehension and working memory in students with PDC suggest that additional factors may contribute to explain the poor outcomes in the comprehension measure. Furthermore, the assessments were carried out as group assessments requiring written instead of oral responses. In some cases, it would have been preferable to use oral responses, for example in assessments of listening comprehension and verbal fluency, to minimize taxing other cognitive processes than the target skill. This needs to be considered when interpreting our findings.

Thirdly, in line with Catts et al. (2006), we applied a more lenient cut-off (*z* ≤ −0.70) than commonly used to identify clinical populations (*z* ≤ −1.00), such as students with dyslexia and developmental language disorder. Thus, our sample of students with PD, PC and PDC included a broader range of reading skills. By including students with word recognition and comprehension skills that would have fallen in the lower end of the typical range with the lower and more commonly applied cut-off, our sample is likely more indicative of the students that teachers meet in their classrooms on a daily basis rather than of the students in a clinical sample. The more lenient cut-off means that our findings may not be fully comparable to other studies and explain why our findings, in some respects, diverge from previous research. However, despite the more lenient cut-off, our findings do converge to a large extent with previous studies, providing additional support and extending previous research to a wider population of students.

Despite these limitations, the longitudinal approach has provided valuable knowledge about the concurrent language and cognitive abilities and the retrospective and prospective reading skills among upper school students with different reading profiles, that have implications for the teaching and learning of students with different reading profiles.

## Data availability statement

The datasets presented in this article are not readily available due to ethical considerations. Requests to access the datasets should be directed to CW, christian.waldmann@lnu.se.

## Ethics statement

The project was assessed by the Regional Ethical Review Board in Umeå, Sweden, as not falling under the Swedish Ethical Review Act (no intervention, no sensitive personal information, no collection of biological material) (dnr 2015/334-331Ö). The board provided a formal statement with recommended amendments that were followed. Written informed consent for participation in this study was provided by parents and students.

## Author contributions

CW: Conceptualization, Formal analysis, Funding acquisition, Investigation, Methodology, Project administration, Resources, Validation, Visualization, Writing—original draft, Writing—review & editing. ML: Conceptualization, Formal analysis, Funding acquisition, Investigation, Methodology, Project administration, Resources, Validation, Visualization, Writing—original draft, Writing—review & editing.
